# Effect of late-onset hemorrhagic cystitis on PFS after haplo-PBSCT

**DOI:** 10.1515/med-2021-0368

**Published:** 2021-10-05

**Authors:** Hailong Yuan, Gang Chen, Jianhua Qu, Ruixue Yang, Maria Muhashi, Gulibadanmu Aizezi, Ming Jiang

**Affiliations:** Hematology Center, First Affiliated Hospital of Xinjiang Medical University, Xinjiang Institute of Hematology, Urumqi 830054, China; Hematology Center, First Affiliated Hospital of Xinjiang Medical University, Xinjiang Institute of Hematology, No. 137 Liyushan South Road, Urumqi 830054, China

**Keywords:** hemorrhagic cystitis, allo-genetic hematopoietic stem cell transplantation, haploid, progression-free survival

## Abstract

**Introduction:**

This study is to investigate the effect of late-onset hemorrhagic cystitis (LOHC) on progression-free survival (PFS) of patients after haploidentical peripheral blood hematopoietic stem cell transplantation (haplo-PBSCT).

**Methods:**

This retrospective study enrolled 74 patients with hematological malignancies treated with a myeloablative conditioning regimen and haplo-PBSCT. The effect of LOHC on PFS was studied in terms of HC occurrence, grade, disease type, duration, onset time, gender, and age.

**Results:**

There were 28 patients with LOHC, and no case was with early-onset HC. The cumulative incidence of LOHC was 37.8% (95% CI: 26.9–48.7%). The 2-year expected PFS of 74 patients and 34 AML patients was not significantly different between LOHC patients and patients without HC (*P* > 0.05). Among 27 ALL patients, the 2-year expected PFS of LOHC patients was 75%, significantly higher than patients without HC (54.2%) (*P* < 0.05). The 2-year expected PFSs of patients with mild LOHC and severe LOHC were 69.8 and 77.8%, respectively (*P* > 0.05). Similarly, the onset time, duration, age, and gender of LOHC patients did not show significant effects on PFS (*P* > 0.05).

**Conclusions:**

After haplo-PBSCT, LOHC has a significant effect on the PFS of ALL patients. The HC grade, duration, onset time, gender, and age have no significant effect on PFS.

## 1 Introduction

Allo-genetic hematopoietic stem cell transplantation (allo-HSCT) is currently one of the effective treatments for hematological malignancies. Hemorrhagic cystitis (HC) is one of the common and serious complications of allo-HSCT. It has been reported that HC is mainly the result of diffuse inflammation and vascular damage of the bladder mucosa caused by immunosuppression and opportunistic infections [1]. Studies have shown that the incidence of HC is 7–68% and the incidence of severe HC is 29–44% [2,3,4]. According to the time of occurrence, it can be divided into early-onset hemorrhagic cystitis (EOHC) and late-onset hemorrhagic cystitis (LOHC). EOHC occurs within 28–72 h of pretreatment, while LOHC occurs 72 h after pretreatment. LOHC is a dangerous and life-threatening disease, which is often associated with the reactivation of various viruses (such as adenovirus, polyoma virus, and BK polyomavirus (BKV)) [5]. Its clinical symptoms are mostly hematuria (manifested from microscopic hematuria, mild urine symptoms to severe bleeding and blood clots), frequent urination, and urgent urination, painful urination, and other bladder irritation symptoms. Mild HC can heal spontaneously while severe HC can cause urinary tract obstruction or renal insufficiency [6].

Kerbauy et al. [7] reported that HC did not significantly affect long-term survival but severe HC would reduce the long-term survival after transplantation. However, it has been shown that BKV-associated HC is a main cause of morbidity after allo-HSCT and is associated with prolonged hospitalization [8,9]. BKV-associated HC is also reported to be associated with an increased risk of treatment-related mortality but limited impact on overall survival [10,11]. Thus, there is controversy whether HC affects progression-free survival (PFS) in patients after allo-HSCT. In our transplantation center, we performed haploidentical peripheral blood hematopoietic stem cell transplantation (haplo-PBSCT) and ATG-based myeloablative conditioning (MAC) regimen on patients with malignant hematological diseases. However, the effect of HC on PFS of patients after MAC regimen haplo-PBSCT is unclear.

In this study, we aim to investigate whether HC has a significant impact on the PFS of patients after haplo-PBSCT. We retrospectively analyzed the data of 74 patients with malignant hematological diseases who received haplo-PBSCT from 2017 to 2019 in our transplantation center. The effects of the occurrence of HC, the severity of HC, time of occurrence, and duration of HC on the PFS of patients was studied. The results will be helpful for the improvement of the diagnosis and treatment of HC and improve the long-term survival of patients.

## 2 Materials and methods

### 2.1 Patients

A total of 74 patients with hematological malignancies, who accepted the myeloablative pretreatment program and haplo-PBSCT in the transplantation center during January 1, 2017 and December 31, 2019, were enrolled in this study. There were 44 male and 30 female patients. The median age was 27 years old (5–54 years old). Disease types included 34 cases of acute myeloid leukemia (AML) (32 cases of high risk and 2 cases of intermediate risk), 27 cases of acute lymphocyte leukemia (ALL) (20 cases of high risk and 7 cases of standard risk, including two children, 5 and 14 years old), 7 cases of myelodysplastic syndrome (MDS) (IPSS-R, 1 case of high risk, 5 cases of high risk and 2 cases of intermediate risk), and 5 cases of chronic myeloid leukemia (CML) (3 cases of accelerated phase and 2 cases of T315I). The inclusion criteria were as follows: (1) the age of patient ranged from 4 to 55 years old; (2) patients had no obvious damage to important organs such as heart, lungs, and kidneys; and (3) patients with malignant hematological tumors had indications for hematopoietic stem cell transplantation. Exclusion criteria were as follows: (1) patients aged younger than 4 years old or older than 55 years old; (2) patients with an active infection that was difficult to control; (3) patients with heart, lung, and kidney damage and severe liver damage (Child grade B–C); (4) pregnant patients; and (5) patients with mental illness. Written informed consent was obtained from every patient and the study was approved by the ethics review board of Xinjiang Medical University (ethical approval number: 20180622-10).

### 2.2 Follow-up

The patients were followed up by four experienced specialists through phone or outpatient visits. The start time of the follow-up was after hematopoietic stem cell transplantation. The deadline for follow-up was June 30, 2020. The median follow-up time was the median survival time of all patients at the end of the follow-up. The PFS was defined as the survival time of patients (in remission) at the end of the follow-up. All patients were followed up regularly without censored data.

### 2.3 Data collection

The main data collected in the study included type of disease, gender, and age of patients with haplo-PBSCT, gender and age of donor, transplant pretreatment plan, MNC infusion volume, CD34^+^ cell infusion volume, GVHD prevention, HC prevention, and HC occurrence severity, HC occurrence time and duration, virus infection, HC treatment method and outcome, patient follow-up time and survival status, patient’s cause of death, etc.

### 2.4 MAC conditioning regimen

For patients with haploid transplantation, an ATG-based Ara-C + Bu/Cy regimen was used. Intravenous drips of cytarabine (Ara-C) at a dose of 2–4 g m^2^ day^−1^ (−9 to −8 days), busulfan (Bu) at a dose of 3.2 mg kg^−1^ day^−1^ (−7 to −5 days), cyclophosphamide (Cy) at a dose of 1.8 g m^−2^ day^−1^ (−3 to −2 days), and rabbit anti-human ATG at a dose of 2.5 mg kg^−1^ day^−1^ (−4 to −1 days) were administered.

### 2.5 Prevention of GVHD

The program was based on CsA or Tac + short-term MTX + MMF + Glu. CsA was administered at an initial dosage of 2–2.5 mg kg^−1^ day^−1^ intravenously, and at 4–5 mg kg^−1^ day^−1^ orally (−5 to +100 days). Tac was administered at an initial dosage of 0.02 mg kg^−1^ day^−1^ intravenously and at 0.05 mg kg^−1^ day^−1^ orally twice (−5 to +100 days). MMF was administered at 0.5 g p.o. twice a day. MTX was administered at 15 mg m^−2^ day^−1^ (+1 day) and 10 mg m^−2^ day^−1^ (+3, +6, +11 days ivgtt). Anti-CD25 monoclonal antibody (12 mg m^−2^) was administered at 20 mg (ivgtt, q.d.) (+1 and +2 days). After transplantation (+1 to +15 days), dexamethasone was administered (2.5 mg i.v., q12h) and then oral administration of prednisone (+16 to +22 days, 20 mg, q.o.d.; +23 to +30 days, 10 mg, q.o.d.; and then stopped) was performed, which was stopped within 3–4 weeks according to the patients’ condition.

### 2.6 Stem cell mobilization and collection

The donor stem cells from peripheral blood were mobilized by G-CSF (5–10 µg kg^−1^ day^−1^, from −4 days). The 2–3 TBV of peripheral blood stem cells were collected at 01 d and 02 d. Then, the donor’s peripheral blood PBSC was infused via the central vein on the 5th and 6th day after mobilization, setting mononuclear cells (MNC) 12–20 × 10^8^ kg^−1^ or CD34^+^ cells ≥8 × 10^6^ kg^−1^. If the donor’s weight was less than that of the patient over 5 kg, it is estimated that the required number of MNC or CD34^+^ cells will not be reached, and thus the stem cell collection can be carried out for 3 days.

### 2.7 Virus infection prevention

Pretreatment of virus infection was performed by administering ganciclovir 250 mg (ivgtt b.i.d, −7 to −1 days) and acyclovir 250 mg (ivgtt b.i.d, +1 day). After the patient’s neutrophils increased to more than 0.5 × 10^9^ L^−1^, the patient was administered 250 mg ganciclovir (ivgtt b.i.d, +15 days), which was continuously used for 2 weeks according to the results of the blood routine.

### 2.8 HC grading

HC grading was defined as follows: Grade I, microscopic hematuria; Grade II, gross hematuria; Grade III, gross hematuria with blood clots; and Grade IV, urethral obstruction with gross hematuria and blood clots. Grades I and II were considered mild, and Grades III and IV were considered severe [12,13].

### 2.9 HC prevention

During the period of Cy administration, the patients were given a high-dose fluid infusion for 24 h to alkalize urine and unify diuresis, and mesna (sodium thioethanesulfonate) was added to prevent HC. In detail, the high-dose fluid infusion was intravenously administered at a dose of 100–120 mL kg^−1^ day^−1^ for 24 h consecutively. To alkalizate urine, the amount of sodium bicarbonate was 0.5% of the total fluid infusion. Diuretic and furosemide injection was given 20 mg/time, once every 6 h (the dosage and frequency were adjusted according to the patient’s symptoms and electrolytes). Meanwhile, potassium was supplemented. The amount of potassium chloride was 1.5% of the total fluid supplement. The dosage of mesna was 1.2 times that of CTX. Its first dose was 20% of CTX, and it was used at the same time as CTX. The remaining mesna was intravenously administered continuously for 24 h.

### 2.10 Related examinations for HC

When HC occurred in the patients, urinary tract ultrasound, gynecological ultrasound for female patients, multiple urine routines, urinary bacterial and fungal cultures were performed, and cystoscopy was also carried out if necessary.

### 2.11 Virus detection for HC

Blood CMV virus antibody and DNA, blood BK and JC virus, urine CMV, BK, and JC virus were detected. Meanwhile, blood and urine BK and JC viruses were tested for the patients without HC during the same period.

### 2.12 General treatment of HC

Once the patient was diagnosed, fluid infusion, diuresis, and alkalization of urine were given. Ribavirin or acyclovir was used empirically for antiviral therapy. For the patients with BKV and other viruses detected in the urine, after assessing the condition, immunosuppressants (especially glucocorticoids) were reduced or stopped, and acyclovir or cyclovir for antiviral therapy were actively used. If there were decreased blood cells, ganciclovir could be replaced with foscarnet sodium. Human immunoglobulin was given at a dose of 0.4 g per kilogram of body weight for 3–4 days. For the patients without virus infection, on the basis of fluid infusion and treatment with human immunoglobulin, etc., if the patients had no significant improvement within 7–10 days, intravenous administration of methylprednisolone or dexamethasone could be considered for the patients with severe HC.

### 2.13 Adipose-derived mesenchymal cell (ADSC) treatment for severe HC

For the patients with severe HC, if no improvement was achieved after more than one month of comprehensive treatments (such as antiviral therapy, rehydration, and diuresis), ADSCs were then used for the adjuvant therapy. The dosage of ADSCs was 1 × 10^6^ kg^−1^ for each infusion (once a week), followed by intravenous injection with 5 mg dexamethasone. During the ADSC infusion, the patient’s blood pressure, heart rate, respiration, body temperature, and with or without chilling and/or dyspnea were closely monitored. The patients received urine routine tests every day, and the patient’s symptoms and signs were recorded in detail. After 3 infusions of ADSCs, if there was still no significant improvement in the assessment of symptoms, the infusion would be stopped.

### 2.14 Evaluation of HC treatment

There were no standard efficacy evaluation criteria for HC treatment currently. Based on the previous experience of HC diagnosis and treatment in our center, HC efficacy criteria were set as follows: (1) Cure – the symptoms of frequent urination, urgency, and dysuria disappeared; the urine routine was normal for 7 consecutive days. (2) Significantly effective – severe HC was reduced to mild HC (Grade I–II). (3) Effective – symptoms such as frequent urination, urgency, dysuria, etc., were alleviated; the urinary red blood cell count was reduced by more than 50%, and Grade IV HC was reduced to Grade III HC. (4) Ineffective –  the patient’s symptoms and laboratory tests did not improve.

### 2.15 Statistical analysis

All statistical analysis was performed using SPSS software (Version 25.0, IBM Inc., New York, USA). Measurement data of non-normal distribution were expressed as median (interquartile range 25%, 75%) and compared with *t*-test. Counting data was presented as *n* (%) and analyzed with the Chi-square test. The cumulative incidence of HC and PFS was analyzed by Kaplan–Merier survival analysis and Log-rank test. A *P* value less than 0.05 was considered statistically significant.

## 3 Results

### 3.1 Evidence of hematopoietic reconstruction and engraftment

In the 74 haplo-PBSCT patients, the stem cells were all implanted. The median number of transplanted MNC was 12.3 (8.2–18.9) × 10^8^ kg^−1^, and the median of CD34^+^ cells was 7.8 (4.02–15.4) × 10^6^ kg^−1^. The median time for neutrophils ≥0.5 × 10^9^ L^−1^ was 16 (12–32) days, and the median time for BPC ≥ 20 × 10^9^ L^−1^ without platelet transfusion was 17 (14–34) days. After transplantation, all patients underwent multiple HLA typing, and the chromosome or red blood cell antigen or whole blood, T, B, and NK cell chimeric rates showed that the patient’s hematopoietic function was replaced by the donor and formed a complete chimera.

### 3.2 Occurrence of HC

**Figure 1:**
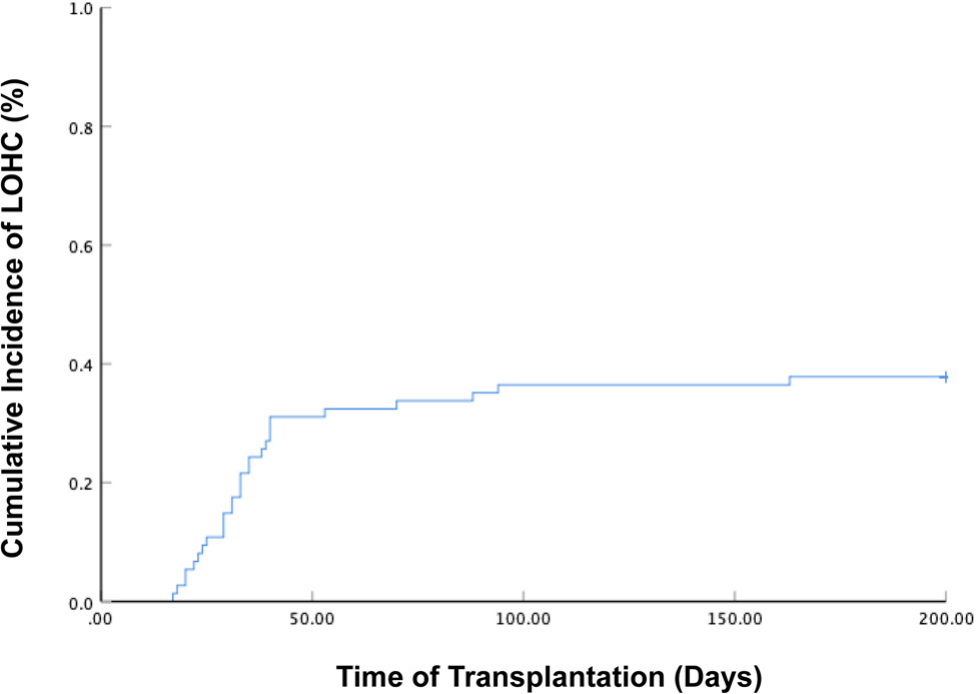
The cumulative incidence of LOHC in the 74 patients after haplo-PBSCT was 37.8% (95% CI: 26.9–48.7%).

HC occurred in 28 of the 74 enrolled patients. All of the 28 cases with HC were LOHC. There were 14 female and 14 male patients. The median onset time was 33 (17–163) days after transplantation, and the median duration was 19 (5–70) days. Among them, there were 2 cases of Grade I, 14 cases of Grade II, 10 cases of Grade III, and 2 cases of Grade IV. LOHC occurred in 9 of 34 patients with AML (1 case of Grade I, 4 cases of Grade II, and 4 cases of Grade III); in 13 of 27 patients with ALL (1 case of Grade I, 5 cases of Grade II, 6 cases of Grade III and 1 case of Grade IV); in 4 of 8 cases of MDS patients (Grade II); and, in 2 of 5 cases of CML patients (1 case of Grade II and 1 case of Grade IV). The cumulative incidence of LOHC in the 74 patients was 37.8% (95% CI: 26.9–48.7%) (Figure 1).

### 3.3 HC and virus infection

Among the 28 patients with HC, 19 received blood and urine BK and JC tests. Urine BK virus was positive in 12 patients, with the lowest virus level of 1.8 × 10^5^ copies/mL and the highest virus level of 8.96 × 10^5^ copies/mL. This indicates that the occurrence of HC may be closely related to the urine BK virus. Only 1 patient was positive for blood BK-DNA, and the remaining 18 patients were negative for blood BK-DNA. Five patients were positive for BK and JC virus in urine but no patient was positive only for JC. The 28 cases of HC patients were negative for CMV in blood and urine and negative for Epstein-Barr virus.

### 3.4 Relapse, death, and survival

The median follow-up time was 14 (4–42) months. Twenty patients died, of which 12 died after recurrence, 5 from severe pneumonia, 1 from intracranial infection, 1 from sepsis, and 1 from sudden death. Relapse was still the main cause of death. No patient died directly from GVHD and no patient died directly or indirectly from LOHC.

### 3.5 PFS rate of patients with/without LOHC and patients with different LOHC grades

**Figure 2:**
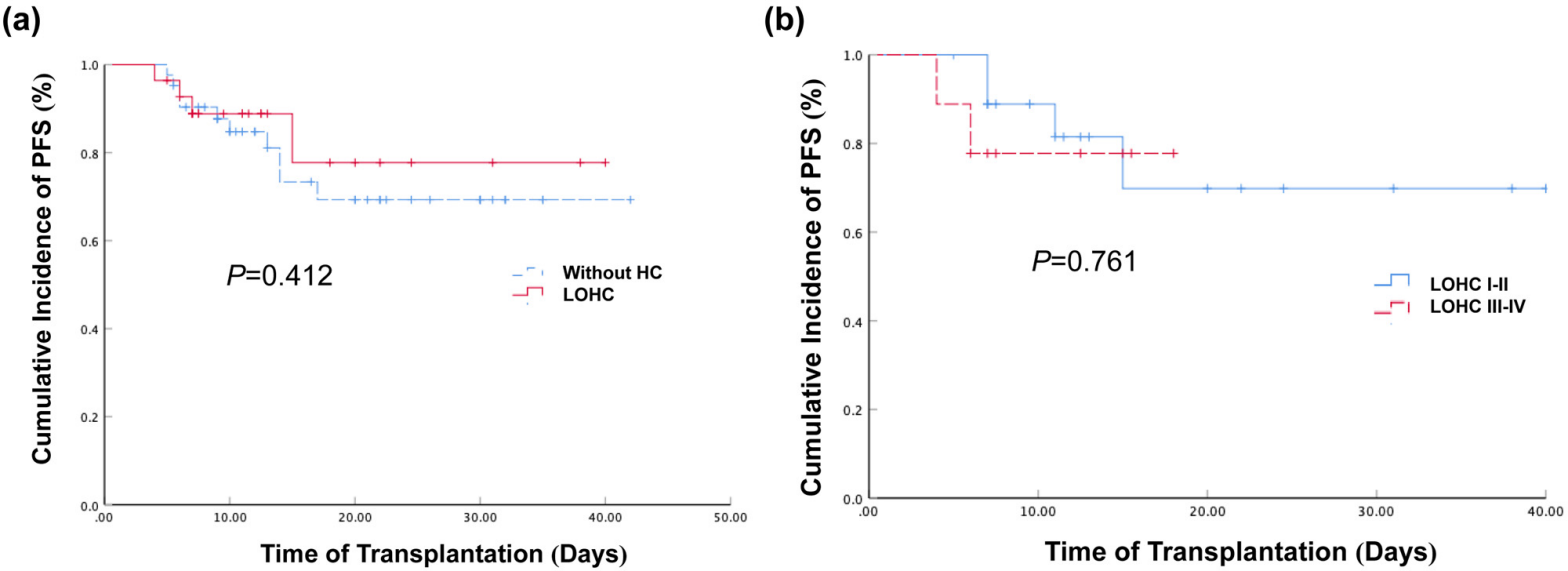
The cumulative incidence of PFS in patients with LOHC/without HC and patients with different LOHC grades. (a) The cumulative incidence of PFS in patients with LOHC and those without HC after haplo-PBSCT were 69.3% (95% CI: 52.6–85.9%) and 77.7% (95% CI: 59.9–95.5%), respectively. The *P* value was 0.412. (b) The cumulative incidence of PFS in LOHC I–II and LOHC III–IV groups after haplo-PBSCT were 69.8% (95% CI: 43–96.6%) and 77.8% (95% CI: 50.6–105%), respectively. The *P* value was 0.761.

The 2-year expected PFS rate of 28 patients with LOHC was 69.3% (95% CI: 52.6–85.9%) and that of 46 patients without HC was 61.9% (95% CI: 45.6–78.2%). The PFS of HC patients was higher than that of the patients without HC but the difference was not statistically significant (*P* > 0.05, Figure 2a).

Among the 28 LOHC patients, 16 were mild LOHC (Grades I–II) and 12 were severe LOHC (Grade III–IV). The 2-year expected PFS rates of mild and severe LOHC patients were 69.8% (95% CI: 43–96.6%) and 77.8% (95% CI: 50.6–105%), respectively. The PFS of the mild LOHC group was lower than that of the severe LOHC group but the difference was not statistically significant (*P* > 0.05, Figure 2b). The grading of LOHC did not show a significant impact on the PFS of patients after transplantation.

### 3.6 Effect of LOHC on PFS in different disease types

**Figure 3:**
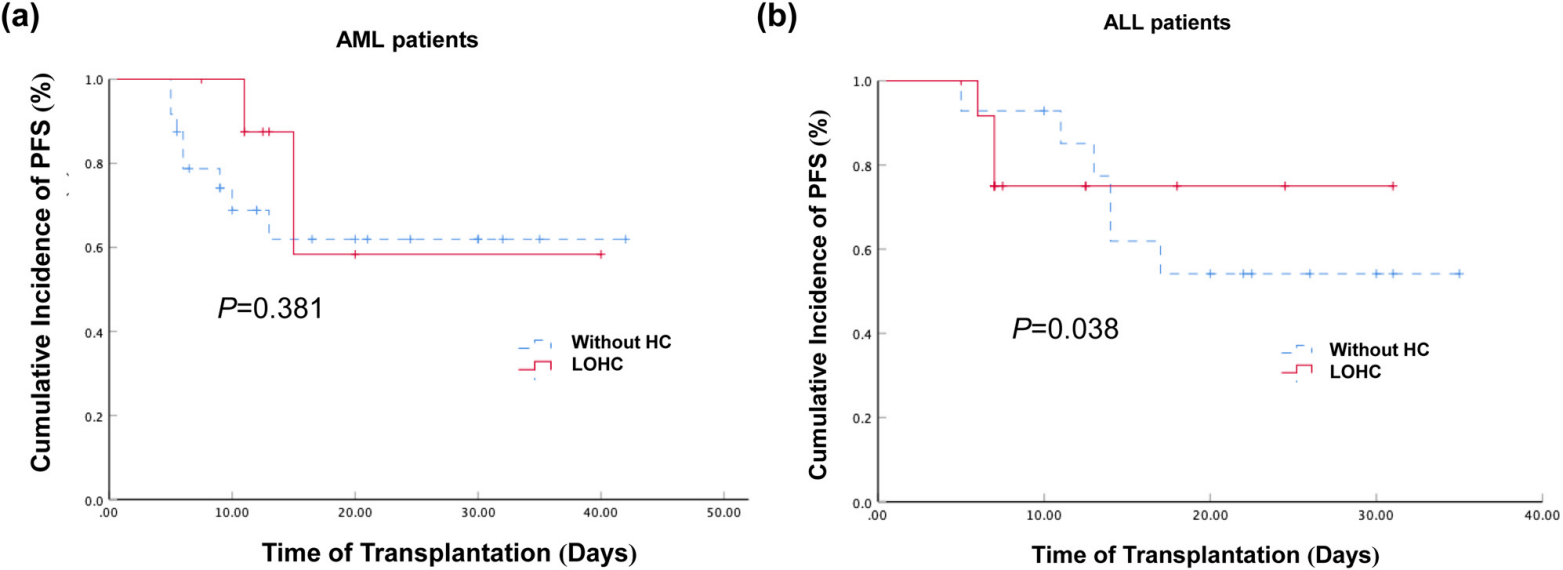
Effect of LOHC on the cumulative incidence of PFS in patients with AML and ALL. (a) The cumulative incidence of PFS of AML in patients with LOHC and those without HC after haplo-PBSCT were 61.9% (95% CI: 40.3–83.5%) and 58.3% (95% CI: 35.3–81.3%), respectively. The *P* value was 0.381. (b) The cumulative incidence of PFS of ALL in patients with LOHC and those without HC after haplo-PBSCT were 54.2% (95% CI: 31.7–76.7%) and 75% (95% CI: 51.5–98.5%), respectively. The *P* value was 0.038.

Among the 34 AML patients, the 2-year expected PFS rate of the 25 patients without HC was 61.9% (95% CI: 40.3–83.5%) and that of the 9 patients with LOHC was 58.3% (95% CI: 35.3–81.3%) (Figure 3a). However, there was no significant difference between them (*P* > 0.05). This suggests that the occurrence of LOHC does not significantly affect the long-term survival of AML patients.

For the 27 ALL patients, 14 were without HC (6 deaths) and the 2-year expected PFS rate was 54.2% (95% CI: 31.7–76.7%), while the 2-year expected PFS rate for the 13 patients with LOHC (3 deaths) was 75% (95% CI: 51.5–98.5%). The PFS of the ALL patients with LOHC was higher than those without HC, and the difference was statistically significant (*P* < 0.05, Figure 3b). This suggests that ALL patients with LOHC have better long-term survival.

Four of the eight MDS patients developed LOHC and none of them survived until the end of follow-up. LOHC had no significant effect on the PFS of patients with MDS. All 5 patients with CML survived, among whom 2 patients were with LOHC and 3 patients were without HC. HC had no significant effect on the PFS of patients with CML.

### 3.7 Effect of LOHC duration and onset time on PFS

**Figure 4:**
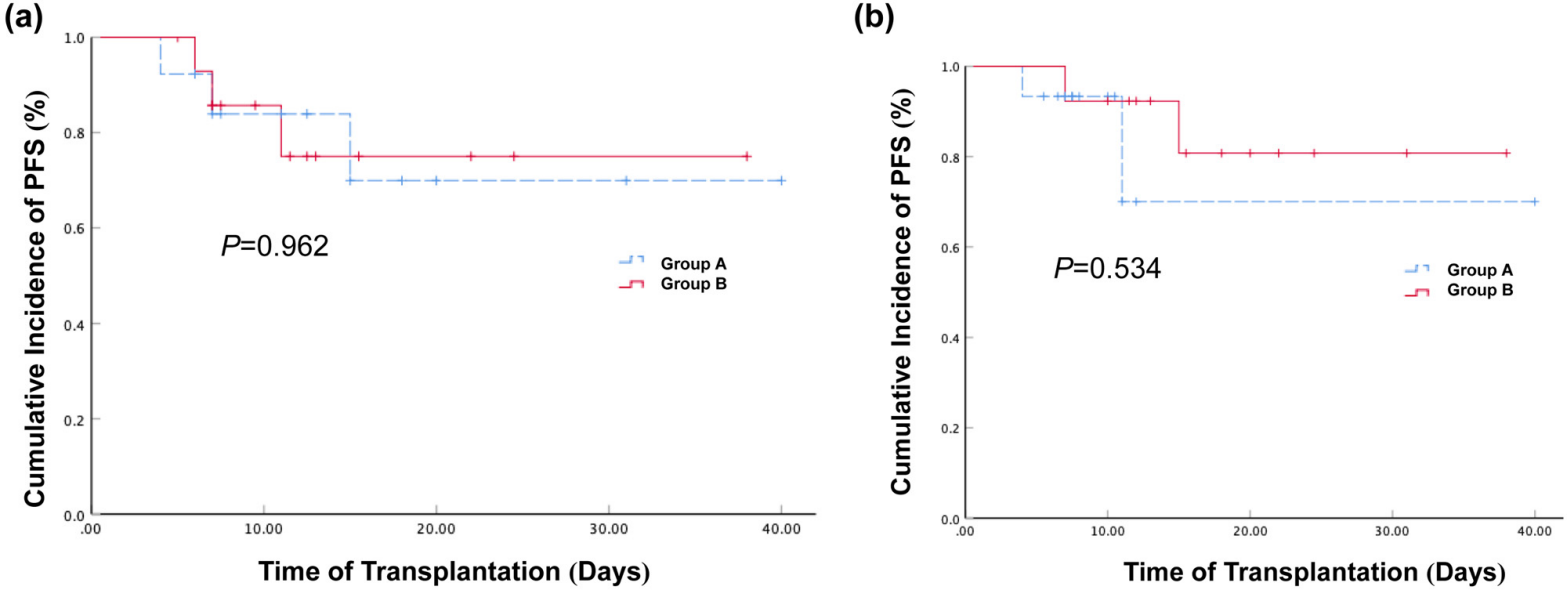
Effect of LOHC duration and onset time on the cumulative incidence of PFS. (a) The cumulative incidences of PFS in patients with LOHC duration for more than 19 consecutive days and patients with LOHC duration for less than 19 days after haplo-PBSCT were 69.9% (95% CI: 39–100.8%) and 75% (95% CI: 49.7–100.9%), respectively. The *P* value was 0.962. (b) The cumulative incidence of PFS in LOHC patients with LOHC median onset time for less than 33 days and longer than 33 days after haplo-PBSCT were 70% (95% CI: 29.2–110.8%) and 80% (95% CI: 55.2–105.8%), respectively. The *P* value was 0.534.

The median duration of LOHC in the 28 LOHC patients was 19 days after transplantation, among whom 13 patients were with a duration of more than 19 days and 15 patients were with a duration of less than 19 days. The 2-year expected PFSs of the patients with a duration of more and less than 19 days were 69.9% (95% CI: 39–100.8%) and 75% (95% CI: 49.7–100.9%), respectively (Figure 4a). No significant difference was observed between them (*P* > 0.05).

The median onset time of the 28 LOHC patients was 33 days after transplantation. The 2-year expected PFS of the 15 patients whose LOHC occurred at less than 33 days was 70% (95% CI: 29.2–110.8%), which was not significantly different from that in the 13 patients whose LOHC occurred at more than 33 days (80% (95% CI: 55.2–105.8%), *P* > 0.05, Figure 4b). These indicate that the duration and onset time of LOHC do not show a significant impact on the survival of patients after transplantation.

### 3.8 Effect of gender and age of LOHC patients on PFS

**Figure 5:**
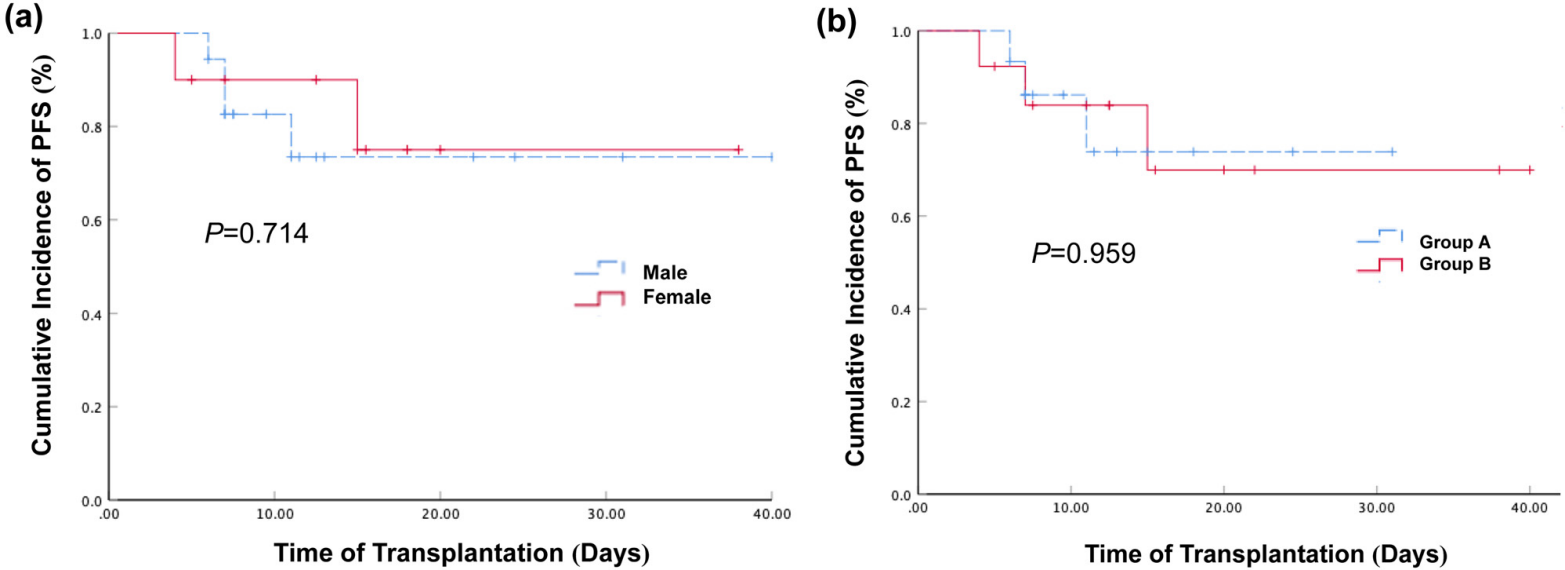
Effect of gender and age of LOHC patients on the cumulative incidence of PFS. (a) The cumulative incidence of PFS in male LOHC and female LOHC patients after haplo-PBSCT were 73.5% (95% CI: 50.2–96.8%) and 75% (95% CI: 44.1–105.9%), respectively. The *P* value was 0.714. (b) The cumulative incidence of PFS in LOHC onset age younger than 27 years old and LOHC onset age older than 27 years old after haplo-PBSCT were 73.8% (95% CI: 46.8–100.8%) and 69.9% (95% CI: 39.52–100.28%), respectively. The *P* value was 0.959.

Among the 28 LOHC patients, 18 were male and 10 were female. The two-year expected PFSs of the male and female LOHC patients were 73.5% (95% CI: 50.2–96.8%) and 75% (95% CI: 44.1–105.9%), respectively. The PFS of the male LOHC patients was slightly lower than that of the female patients but the difference was not statistically significant (*P* > 0.05, Figure 5a).

The median age of the 28 LOHC patients was 27 years old (5 to 54 years), among whom 15 patients were younger than 27 years old and 13 patients were older than 27 years old. The 2-year expected PFSs for the patients younger than 27 years old and older than 27 years old were 73.8% (95% CI: 46.8–100.8%) and 69.9% (95% CI: 39.52–100.28%), respectively (Figure 5b). The two-year expected PFS of the patients younger than 27 years old was higher than that of the patients over 27 years old but the difference was not statistically significant (*P* > 0.05). These results suggest that the genders and ages of LOHC patients have no significant impact on survival after transplantation.

## 4 Discussion and conclusion

HC is a common complication after allo-HSCT, caused by many factors. Some studies [11,14] indicate that ADV infection is a major pathogenic factor of LOHC after allo-HSCT. Some studies have also shown that the occurrence of LOHC is related to CMV, influenza virus, etc. [15,16]. At present, it is generally believed that the BK virus may be the main reason for the occurrence of HC [17,18,19]. The pretreatment regimen, the degree of HLA differences between donors and recipients, and the source of stem cells may all be potential risk factors for the occurrence of HC [20,21,22]. Mukherjee et al. [22] further evaluated the clinical variables related to the occurrence of HC and found that haploid transplantation was the main risk factor for its occurrence. Lu et al. [23] showed that the occurrence of HC during non-*in vitro* depletion of T cell haploids was significantly higher than that of homologous transplantation and believed that ATG was related to the occurrence of HC. Kerbauy et al. [7] indicated that the use of ATG during pretreatment significantly increased the BKPyV infection-associated incidence of HC. A recent study by Mo et al. [24] showed that the cumulative incidence of HC after haplo-HSCT in 149 cases of ATG-based MAC was 29.8% and the cumulative incidence of severe (Grades III–IV) HC was 10.9%. This study used ABuCy as a myeloablative pretreatment program based on ATG and used only peripheral blood as a source of stem cells. For acute leukemia-based malignant hematological diseases, it could reduce the impacts of various factors, such as the types of diseases, pretreatment programs, and ATG dosage, on the occurrence of HC.

The impact of LOHC on survival after transplantation is not clear but recent studies have shown that although LOHC associated with BK virus infection increases the risk of transplant-related mortality, it has a limited impact on overall survival [8,9,25,26]. Arai et al. [27] studied 249 (MAC146 cases) allo-SCT patients for 14 years, and a total of 47 cases of HC occurred, with a cumulative incidence of 21.4%. There was no statistical difference in overall survival (OS) between the patients with and without HC, but the OS of severe HC (Grade III) patients was significantly reduced compared with moderate (Grade I–II) and non-HC patients. Lunde et al. [10] studied 1,321 patients (663 MAC patients) who were transplanted continuously for 9 years. Totally, 219 patients developed HC with an incidence rate of 16.6%. The 1-year expected OS of HC patients and non-HC patients was 63 and 66%, respectively. The difference was not statistically significant, and the severity of HC (Grades I–II and III–IV) did not show a significant impact on OS. Kerbauy et al. [7] studied 133 patients with allo-HSCT, and 36 patients developed HC related to BK virus infection. The 1-year cumulative HC incidence was 27.3%. Compared with patients without HC, the occurrence of HC significantly affected the survival of patients. Among them, Grade III–IV HC significantly affected patient survival, while Grade I–II HC had no significant effect. Therefore, HC has no obvious effect on the survival of allo-HSCT patients overall but severe HC may affect the survival of patients. In this study, the occurrence of LOHC had no obvious effect on PFS and the severity of LOHC had no significant effect on PFS either. Among the ALL patients, the PFS of 13 patients with LOHC was significantly higher than that of 14 patients without HC. However, the underlying reason for this is unclear. Further studies are needed to verify this result.

We also found that the different LOHC occurrence time, LOHC duration, patient age, and gender of 28 LOHC patients did not significantly affect the PFS of the patients, suggesting that LOHC may not be a complication that mainly affects the PFS of patients after haplo-HSCT. For the 28 patients, no renal insufficiency and death were directly caused due to the degree and duration of LOHC, even for the patients with severe LOHC. These results indicate that LOHC has no obvious impact on the PFS of patients, and no LOHC-related or LOHC treatment-related death occurs, which may be related to the stratified treatment of LOHC in our center. Based on previous treatment experiences, the stratified treatment ideas for LOHC patients in our center have been summarized as follows: (1) For BK and other virus-related HC patients, Cidofovir is the first-line treatment drug. However, some studies believe that Cidofovir alone does not play a key role in the alleviation of severe HC symptoms caused by the BK virus, and the current efficacy lacks rigorous clinical trials [28,29]. Therefore, in assessing the occurrence of GVHD, reduce or stop glucocorticoids and other immunosuppressants are very necessary. Continuous intravenous injection of human immunoglobulin for 3–4 days to enhance the patient’s nonspecific immunity is also beneficial to the effective control of the virus. (2) Studies have shown that [30,31] acute GVHD is a risk factor for the occurrence of LOHC. Some patients in this group have GVHD before the onset of HC, especially when the virus test is positive at the time of diagnosis and becomes negative after treatment. However, when the HC is still severe, it should be considered that HC may be related to GVHD. For severe HC patients, intravenous infusion of methylprednisolone is used for treatment and the course of treatment is generally 3–5 days. (3) Patients with refractory and prolonged HC are treated with mesenchymal stem cell (MSC) infusion. In recent years, there have been more and more studies using MSCs to treat HC. For example, Ringden and Le Blanc [32] reported that 8 cases of hematuria disappeared completely after intravenous infusion of MSC in 12 patients with HC after HSCT. Baygan et al. [33] treated 11 patients with HC after HSCT with MSC, and the average disappearance time of hematuria was 22 days. In comparison, the average hematuria lasting time was 42 days for the patients with Grade 3 and above HC without MSC treatment, suggesting that MSC may be a feasible new treatment option for HC patients. In the study by Wang et al. [34], 7 of 33 HC patients were treated with MSC. All these 7 patients received at least one MSC infusion and achieved good therapeutic effects. In this study, the 3 cases of LOHC who received MSC therapy were all refractory and prolonged patients. They had been treated with a variety of treatment methods before and none of them were effective. After infusion of ADSC, good clinical effects were achieved, indicating that ADSC is indeed effective for severe LOHC.

In conclusion, after Haplo-PBSCT, the incidence of LOHC is relatively high. In the case of timely diagnosis and effective treatment, LOHC has no significant effect on the overall PFS of patients. The severity, occurrence time, and duration of LOHC also show no significant effect on PFS. Although LOHC will increase the patient’s pain and hospitalization time, it does not affect the patient’s survival.

## Abbreviations

AML: acute myeloid leukemia
ALL: acute lymphocyte leukemia
ADSC: adipose-derived mesenchymal cell
allo-HSCT: allo-genetic hematopoietic stem cell transplantation
BKV: BK polyomavirus
CML: chronic myeloid leukemia
EOHC: early-onset hemorrhagic cystitis
haplo-PBSCT: haploidentical peripheral blood hematopoietic stem cell transplantation
HC: hemorrhagic cystitis
LOHC: late-onset hemorrhagic cystitis
MAC: myeloablative conditioning
MDS: myelodysplastic syndrome
OS: overall survival
PFS: progression-free survival
